# The pharmacokinetics of anthocyanins and their metabolites in humans

**DOI:** 10.1111/bph.12676

**Published:** 2014-06-10

**Authors:** R M de Ferrars, C Czank, Q Zhang, N P Botting, P A Kroon, A Cassidy, C D Kay

**Affiliations:** 1 Department of Nutrition, Norwich Medical School, University of East AngliaNorwich, UK; 2 Department of Chemistry, St Andrews UniversitySt Andrews, Fife, Scotland, UK; 3 Institute of Food ResearchNorwich Research Park, Norwich, UK

**Keywords:** anthocyanins, metabolites, hippuric acid, ferulic acid, vanillic acid

## Abstract

**BACKGROUND AND PURPOSE:**

Anthocyanins are phytochemicals with reported vasoactive bioactivity. However, given their instability at neutral pH, they are presumed to undergo significant degradation and subsequent biotransformation. The aim of the present study was to establish the pharmacokinetics of the metabolites of cyanidin-3-glucoside (C3G), a widely consumed dietary phytochemical with potential cardioprotective properties.

**EXPERIMENTAL APPROACH:**

A 500 mg oral bolus dose of 6,8,10,3′,5′-^13^C_5_-C3G was fed to eight healthy male participants, followed by a 48 h collection (0, 0.5, 1, 2, 4, 6, 24, 48 h) of blood, urine and faecal samples. Samples were analysed by HPLC-ESI-MS/MS with elimination kinetics established using non-compartmental pharmacokinetic modelling.

**KEY RESULTS:**

Seventeen ^13^C-labelled compounds were identified in the serum, including ^13^C_5_-C3G, its degradation products, protocatechuic acid (PCA) and phloroglucinaldehyde (PGA), 13 metabolites of PCA and 1 metabolite derived from PGA. The maximal concentrations of the phenolic metabolites (*C*_max_) ranged from 10 to 2000 nM, between 2 and 30 h (*t*_max_) post-consumption, with half-lives of elimination observed between 0.5 and 96 h. The major phenolic metabolites identified were hippuric acid and ferulic acid, which peaked in the serum at approximately 16 and 8 h respectively.

**CONCLUSIONS AND IMPLICATIONS:**

Anthocyanins are metabolized to a structurally diverse range of metabolites that exhibit dynamic kinetic profiles. Understanding the elimination kinetics of these metabolites is key to the design of future studies examining their utility in dietary interventions or as therapeutics for disease risk reduction.

## Introduction

Despite the growing evidence for the cardioprotective effects of dietary anthocyanins such as cyanidin-3-glucoside (C3G) (Erdman *et al*., 2007; Mink *et al*., 2007; Cassidy *et al*., 2011; 2013), the mechanisms involved remain poorly defined due to a limited understanding of their bioavailability and metabolism. Anthocyanins are reported to have low bioavailability, with the majority of studies recording a recovery of <1% of the ingested anthocyanin dose (Kay *et al*., 2005; Manach *et al*., 2005; McGhie and Walton, 2007). Several previous human feeding studies have explored the absorption, distribution, metabolism and elimination (ADME) of anthocyanins; however, most studies only reported conjugated derivatives [methyl, glucuronide (GlcA), sulfate] of parent anthocyanins as the major metabolites in the circulation post-consumption, with maximum serum concentrations ranging from 1.4 to 547 nM, between 0.5 and 1.5 h (*t*_max_) post-consumption (Kay *et al*., 2004; Manach *et al*., 2005; McGhie and Walton, 2007).

A human study feeding blood orange juice suggested that the phenolic acid degradation product, protocatechuic acid (PCA), was a major metabolite of anthocyanins (Vitaglione *et al*., 2007). In addition, a range of phenolic acids, including vanillic acid (VA), syringic acid, caffeic acid and ferulic acid, have been identified within human serum, following the consumption of an anthocyanin-rich elderberry extract (de Ferrars *et al*., 2014) and bilberry-lingonberry puree (Nurmi *et al*., 2009). However, the complex flavonoid and phenolic profile of the interventions limited the ability to trace the metabolites back to their source. Furthermore, *in vitro* and human studies indicate that such phenolic acids undergo phase II conjugation (Nardini *et al*., 2006; 2009; Woodward *et al*., 2011), suggesting that anthocyanins can undergo extensive metabolism *in vivo*. Recent evidence also suggests that the major metabolites of anthocyanins are likely to be derived from bacterial fermentation and absorption from the colon (McGhie and Walton, 2007; Del Rio *et al*., 2010; Williamson and Clifford, 2010).

The extensive degradation and metabolism of anthocyanins was recently confirmed in our stable isotope-labelled C3G feeding study in which the relative bioavailability of C3G was established as 12.4 ± 1.4%, based on the recovery of the ^13^C-label in the urine and breath (Czank *et al*., 2013), suggesting that the extent of anthocyanin absorption and metabolism had been previously underestimated. Furthermore, given the degradation, short *t*_½_ and low *C*_max_ of the parent anthocyanins, the observed cardiovascular benefits of anthocyanin consumption (Erdman *et al*., 2007; Mink *et al*., 2007; Cassidy *et al*., 2011; 2013) are probably the consequence of the metabolites, which are present within the circulation for significantly longer and at higher concentrations than the parent anthocyanins.

The present study aimed to identify the unique pharmacokinetic profiles of each anthocyanin metabolite following the consumption of a 500 mg oral bolus dose of ^13^C-labelled C3G. The findings from this work will inform the design of future clinical interventions and mechanistic studies exploring the biological activity of anthocyanins.

## Methods

### Chemicals and reagents

**Figure 1:**
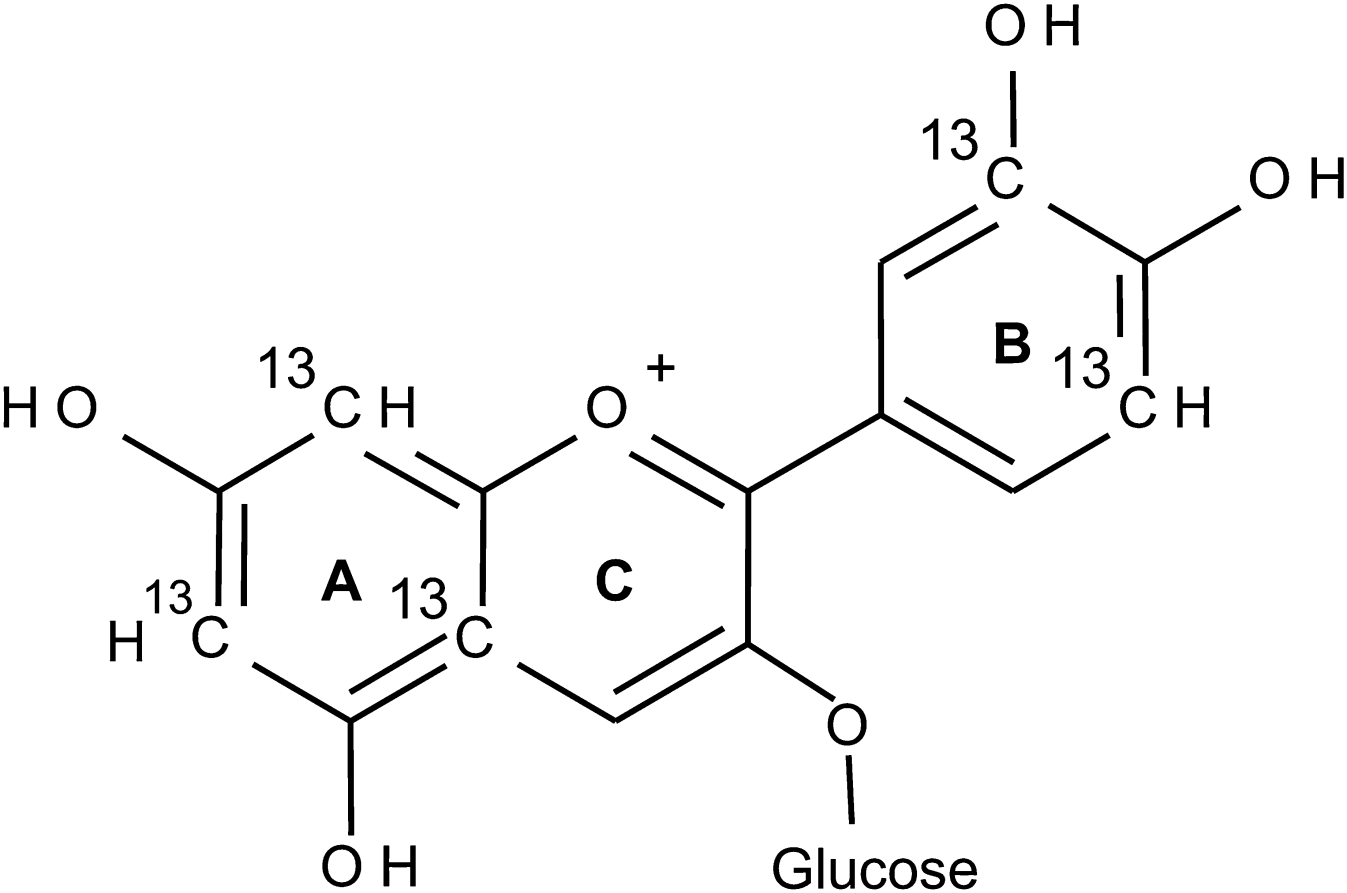
Structure of 6,8,10,3′,5′-^13^C_5_-cyanidin-3-glucoside.

A ^13^C-enriched anthocyanin, 6,8,10,3′,5′-^13^C_5_-C3G (herein referred to as ^13^C_5_-C3G), containing ^13^C at three positions on the A-ring and two positions on the B-ring of the anthocyanin (Figure 1) was synthesized as previously described (Zhang *et al*., 2011). Unlabelled C3G and peonidin-3-glucoside (P3G) were obtained from Extrasynthese (Genay, France) as analytical standards. HPLC-grade acetonitrile was purchased from Fisher Scientific (Loughborough, UK). Phase II conjugates of phenolic acids [PCA-3-GlcA, PCA-4-GlcA, vanillic acid (VA)-4-GlcA, isoVA-3-GlcA, benzoic acid (BA)-4-GlcA, PCA-3-sulfate, PCA-4-sulfate, VA-4-sulfate and isoVA-3-sulfate] were synthesized at St Andrews University, as previously described (Zhang *et al*., 2012). Strata-X™ solid phase extraction (SPE) columns (6 mL, 500 mg), Kinetex pentafluorophenol (PFP) HPLC column (2.6 μM, 100 × 4.6 mm) and SecurityGuard® cartridges (PFP, 4.0 × 2.0 mm) were purchased from Phenomenex (Cheshire, UK). Bond Elute C18 (20 mL, 5 g) SPE columns were from Agilent (Workingham, UK), and Discovery® DSC-18 SPE columns (6 mL, 1 g), Acrodisc PTFE syringe filters (0.45 μm, 13 mm) and all other chemicals were purchased from Sigma-Aldrich (Dorset, UK).

### Clinical design

A detailed description of the clinical design was previously published (Czank *et al*., 2013). Briefly, a single 500 mg oral bolus dose of isotopically labelled C3G (^13^C_5_-C3G) was fed to eight healthy male participants (body mass index, 18.5–30.5 kg·m^–2^; aged 18–45 years) after an overnight fast (>8 h). Participants provided blood (baseline, 0.5, 1, 2, 4, 6, 24, 48 h), urine (individual voids between 0 and 6 h, total voids between 6–24 and 24–48 h), breath (baseline, 0.5, 1, 2, 4, 6, 24, 48 h) and faecal samples (all voids between 0–6, 6–24 and 24–48 h). The study was conducted at the Clinical Research and Trials Unit at the University of East Anglia according to the principles expressed in the Declaration of Helsinki and was approved by the Cambridgeshire 3 Research Ethics Committee (REC ref: 10/H0306/42) and registered at clinicaltrials.gov as NCT01106729.

### Sample extraction and analysis

Serum, urine and faecal samples were extracted by SPE and analysed by HPLC-ESI-MS/MS as recently described (Czank *et al*., 2013). The extraction efficiency was established as 87 ± 11% (coefficient of variation, CV; 6.8 ± 0.7%) for serum, 88 ± 18% (CV, 7.9 ± 0.8%) for urine and 81 ± 21% (CV, 14.1 ± 1.2%) for faeces (*n* = 3 replicates for each analyte). Sample extracts (5 μL) were injected onto a Kinetex PFP column (Phenomonex) and separated using an Agilent 1200 series HPLC with a QTrap 4000 linear ion trap mass spectrometer (ABSciex, Warrington, UK). The HPLC gradient consisted of 0.1% (v v^−1^) formic acid in water (A) and 0.1% (v v^−1^) formic acid in acetonitrile (B), with a flow rate of 1.5 mL·min^−1^ and gradient consisting of 1% B at 0 min, 7.5% B at 7 min, 7.6% B at 14 min, 10% B at 17 min, 12% B at 18.5 min, 12.5% B at 20 min, 30% B at 24 min and 90% B at 25 min. Metabolite identification was performed by multiple reaction monitoring (MRM) optimized for the detection of pure standards with *m*/*z* of the parent and daughter fragments adjusted to +2,+3,+5 amu to allow identification of the ^13^C-labelled metabolites derived from the B-ring, A-ring or the parent structure respectively. Metabolites were confirmed on the basis of retention time (using authentic and synthesized standards where possible) and three or more ion transitions. The HPLC-ESI-MS/MS method was validated for linearity and precision across all metabolites. Six point standard curves ranging from 1.25 to 20 μM were constructed from analytical standards and injected six times. The precision was established as 1.4–11.0% and linearity of the standard curves was established as *r*^2^ = 0.991–1.000 (CV, 0.2%) across the identified metabolites.

### Pharmacokinetic and data analysis

Pharmacokinetic modelling of metabolites in the serum was performed with the PKSolver ‘add-on’ for Excel 2010 (Microsoft, Mountain View, CA, USA) (Zhang *et al*., 2010) using non-compartmental analysis with the AUC calculated using the trapezoidal rule. Pharmacokinetic parameters were established for each individual analyte and presented as mean ± SEM. Urine samples collected during the first 6 h post-bolus were grouped together to the nearest hour time point (i.e. samples were not pooled but individual concentrations were averaged post-quantification) for graphical representation and to calculate total cumulative recovery. The concentration of metabolites was converted to amount recovered from urine by accounting for the molecular weight of the individual ^13^C-labelled metabolites and the volume of the individual participants void and averaged across participants. Recovery of metabolites in the faeces was established from the molecular weight of the ^13^C-labelled metabolite and the weight of the individual participants void adjusting for faecal water content as previously described (Czank *et al*., 2013). Urine and faecal recovery amounts are presented as mean ± SEM (*n* = 8), unless otherwise stated.

## Results

### Metabolite identification

A total of 35 ^13^C-labelled analytes (including the parent ^13^C_5_-C3G) were identified in the serum, urine and faecal samples collected over 48 h post-consumption. The metabolites identified included the degradation products of C3G [PCA and phloroglucinaldehyde (PGA)], phase I (dehydroxylation, reduction) and phase II (methyl, sulfate, glycine and glucuronyl) conjugates of C3G, PCA and PGA, and probable bacterial metabolites, including carboxylic, phenylacetic and phenylpropenoic acids. Of the 35 analytes identified, six metabolites (two isomers of cyanidin-glucuronide, methyl-cyanidin-glucuronide and three isomers of methyl-cyanidin-3-glucose-glucuronide; Table 1) were tentatively identified as having a neutral loss of 176 *m*/*z* (GlcA) while sharing common daughter ion transitions with C3G or P3G. The remaining 29 metabolites [C3G, P3G, PCA, PGA, hydroxybenzoic acid, BA-4-GlcA, PCA-3-GlcA, PCA-4-GlcA, PCA-3-sulfate, PCA-4-sulfate, VA, isoVA, VA-4-GlcA, isoVA-3-GlcA, VA-4-sulfate, isoVA-3-sulfate, 4-hydroxyphenylacetic acid, 3,4-dihydroxyphenylacetic acid, 4-hydroxybenzaldehyde (4-HBAL), 3,4-dihydroxybenzaldehyde, caffeic acid, ferulic acid (both A- and B-ring derived), hippuric acid, 4-methoxybenzaldehyde, 2-hydroxy-4-methoxybenzoic acid, methyl vanillate and methyl-3,4-dihydroxybenzoate (Table 1)] were confirmed by comparison of retention time and MS/MS fragmentation patterns to that of pure standards.

**Table 1:** HPLC-MS/MS identification (MRM) of ^13^C-labelled cyanidin-3-glucoside (C3G) and its metabolites in the serum, urine and faeces of healthy volunteers (*n* = 8) after the consumption of 500 mg of ^13^C-labelled C3G

Metabolite	Compound identification				Analytical standards
	*R*_t_ (min)	MRM ion transitions (*m*/*z*)^a^	Location	# ^13^C^b^	MS^2^ fragments (*m/z*)
Parent anthocyanins					
Cyanidin-3-glucoside (C3G)	12.6	454/292, 246, 218, 133	Urine, serum, faeces	+5	449/287, 241, 213, 128
Cyanidin-glucuronide	11.7	468/292	Urine	+5	NA
Cyanidin-glucuronide	16.5	468/292	Urine	+5	NA
Peonidin-3-glucoside	16.6	468/306	Urine	+5	463/301
Methyl-cyanidin-glucuronide	16.7	482/306	Urine	+5	NA
Methyl-C3G-glucuronide	5.7	644/306	Urine	+5	NA
Methyl-C3G-glucuronide	8.0	644/306	Urine	+5	NA
Methyl-C3G-glucuronide	9.6	644/306	Urine	+5	NA
Degradants					
Protocatechuic acid (PCA)	4.3	155/111, 93, 83	Urine, serum, faeces	+2	153/109, 91, 81
Phloroglucinaldehyde	7.4	156/155, 128, 110, 86	Urine, serum, faeces	+3	153/151, 125, 107, 83
Metabolites					
3-Hydroxybenzoic acid	8.1	138/95, 67	Urine, faeces	+2	136/93, 65
4-Hydroxybenzoic acid	6.5	138/95, 67	Urine, faeces	+2	136/93, 65
Benzoic acid-4-glucuronide	3.0	317/175, 155, 113, 95	Urine, serum, faeces	+2	315/175, 153, 113, 93
PCA-3-glucuronide	4.3	331/175, 155, 113, 111	Urine, serum, faeces	+2	329/175, 153, 113, 109
PCA-4-glucuronide	3.3	331/175, 155, 113, 111	Urine, serum, faeces	+2	329/175, 153, 113, 109
PCA-3-sulfate	6.5	235/191, 155, 111	Urine, serum, faeces	+2	233/189, 153, 109, 97
PCA-4-sulfate	6.2	235/191, 155, 111	Urine, serum, faeces	+2	233/189, 153, 109, 97
Vanillic acid (VA)	9.5	169/154, 125, 110	Urine, serum, faeces	+2	167/152, 123,108
IsoVA	9.9	171/153, 127, 95	Urine, serum, faeces	+2	169/151, 125, 93, 65
IsoVA-3-glucuronide	6.2	347/175, 171, 154, 113	Urine, serum, faeces	+2	345/175, 169, 152, 113
VA-4-glucuronide	4.9	347/175, 171, 154, 113	Urine, serum, faeces	+2	345/175, 169, 152, 113
IsoVA-3-sulfate	8.8	247/169, 154, 125, 110	Urine, serum, faeces	+2	245/167, 152, 123, 108
VA-4-sulfate	8.6	247/169, 154, 125, 110	Urine, serum, faeces	+2	245/167, 152, 123, 108
4-Hydroxyphenylacetic acid	6.8	154/125, 109, 95	Urine, faeces	+2	152/123, 107, 93, 79
3,4-Dihydroxyphenylacetic acid	4.6	171/127, 111, 97	Urine, faeces	+2	169/125, 109, 95
4-Hydroxybenzaldehyde	8.5	123/110, 94, 67	Urine, serum, faeces	+2	121/108, 92, 65
3,4-Dihydroxybenzaldehyde	5.8	139/110, 94, 83	Urine, faeces	+2	137/109, 92, 81
Caffeic acid	10.4	183/139, 110, 93	Faeces	+2	181/137, 108, 91
Ferulic acid	18.8	195/180, 150, 136	Urine, serum, faeces	+2	193/178, 148, 134
		196/181, 151, 137	Urine, serum, faeces	+3	
Hippuric acid	6.3	179/136, 134, 79	Urine, serum, faeces	+2	177/134 ,132, 77
4-Methoxybenzaldehyde	19.6	169/125, 110, 81	Faeces	+2	167/123, 108, 79
2-Hydroxy-4-methoxybenzoic acid	20.1	155/94, 79, 65	Faeces	+2	153/92,77, 63
		156/95, 80, 66	Urine	+3	
Methyl vanillate	21.4	185/153, 125, 109	Faeces	+2	183/151, 123, 107, 77
Methyl-3,4-dihydroxybenzoate	12.9	170/111, 93	Urine, serum, faeces	+2	168/109, 91

a Mass spectra fragments for ^13^C-labelled metabolites with +2, +3 and +5 *m*/*z*.

b +2, +3 and +5 *m*/*z* refer to metabolites derived from PCA, PGA and cyanidin respectively.

NA, no analytical standard available for confirmation.

### Serum pharmacokinetics

**Figure 2:**
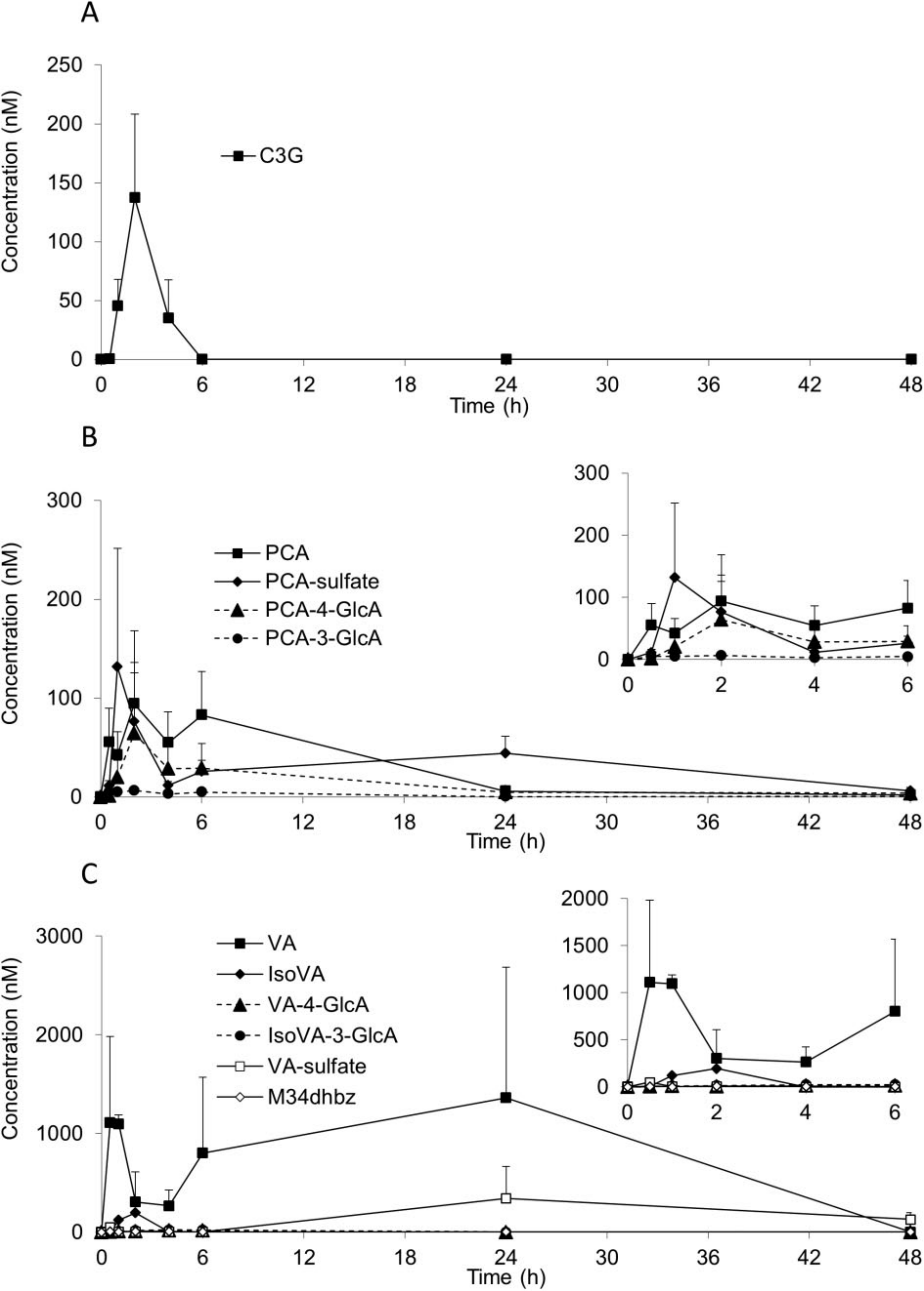
Serum pharmacokinetic profiles of (A) C3G, (B) PCA and its metabolites and (C) methylated PCA and its metabolites, in humans after the consumption of 500 mg ^13^C_5_-C3G in eight healthy male participants. All data are mean ± SEM. C3G, cyanidin-3-glucoside; GlcA, glucuronide; M34dhbz, methyl-3,4-dihydroxybenzoate; PCA, protocatechuic acid; VA, vanillic acid.

**Figure 3:**
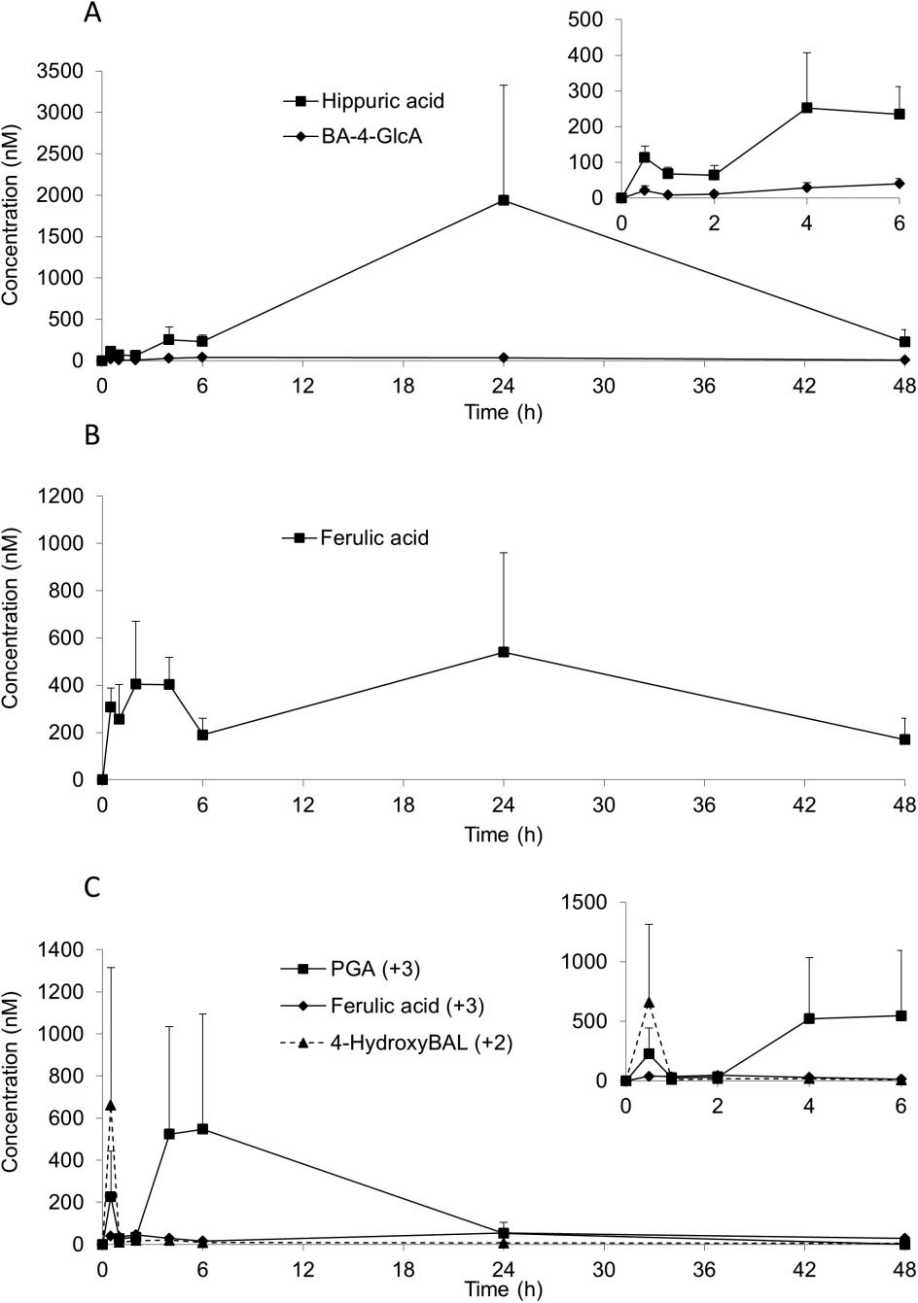
Serum pharmacokinetic profiles of (A) benzoic acid metabolites, (B) propenoic acid metabolites, and (C) A-ring-derived and aldehyde metabolites of cyanidin-3-glucoside in humans after the consumption of 500 mg ^13^C_5_-C3G in eight healthy male participants. All data are mean ± SEM. BA, benzoic acid; BAL, benzaldehyde; GlcA, glucuronide; PGA, phloroglucinaldehyde.

In the serum sample, a total of 17 ^13^C-labelled compounds, comprising C3G, PCA, PGA, 13 derivatives of PCA and 1 derivative of PGA, were detected (Table 2; Figures 2 and 3). The *C*_max_ of the analytes ranged from 11 nM for PCA-3-GlcA (Figure 2B) to 1962 nM for hippuric acid (Figure 3A), with *t*_max_ between 1.8 h for C3G and 30.1 h for VA-sulfate (Table 2). Elimination half-lives (*t*_½_) also ranged from 0.4 h for C3G to 96.5 ± 73.4 h for the A-ring-derived ferulic acid (Table 2). PCA-3-GlcA was the least abundant metabolite present in the serum, whereas hippuric acid was the most abundant metabolite (Table 2).

**Table 2:** Serum pharmacokinetic profiles of cyanidin-3-glucoside (C3G), its degradation products and derived metabolites in humans after the consumption of 500 mg ^13^C-labelled C3G^a^

Metabolite	*n*^b^	*C*_max_ (nM)	*t*_max_ (h)	*t*_1/2_ (h)	AUC_0-48_ (nmol h L^−1^)
Parent anthocyanins					
Cyanidin-3-glucoside	5	141 ± 70	1.8 ± 0.2	0.4	279 ± 170
Degradants					
Protocatechuic acid (PCA)	8	146 ± 74	3.3 ± 0.7	9.9 ± 3.4	1377 ± 760
Phloroglucinaldehyde	4	582 ± 536	2.8 ± 1.1	NQ	7882 ± 7768
Protocatechuic acid derived					
Benzoic acid-4-glucuronide	7	74 ± 20	10.9 ± 3.4	17.1 ± 3.0	1467 ± 489
Methyl-3,4-dihydroxybenzoate	8	12 ± 5	8.4 ± 5.7	21.6 ± 5.9	171 ± 70
PCA-3-glucuronide	5	11 ± 3	2.7 ± 1.0	18.0 ± 15.6	60 ± 38
PCA-4-glucuronide	8	68 ± 61	3.8 ± 0.8	19.4 ± 3.1	618 ± 489
PCA-sulfates^c^	8	157 ± 116	11.4 ± 3.8	31.9 ± 19.1	1180 ± 349
Vanillic acid (VA)	2	1845 ± 838	12.5 ± 11.5	6.4	23 319 ± 20 650
IsoVA	1	195	2.0	NQ	189
VA-4-glucuronide	8	24 ± 4	4.8 ± 0.4	NQ	74 ± 11
IsoVA-3-glucuronide	8	35 ± 5	4.3 ± 0.6	1.6 ± 0.2	103 ± 13
VA-sulfates^c^	4	430 ± 299	30.1 ± 11.4	NQ	10 689 ± 7751
4-Hydroxybenzaldehyde	7	667 ± 653	5.6 ± 3.1	17.9 ± 8.8	663 ± 505
Ferulic acid	7	827 ± 371	8.2 ± 4.1	21.4 ± 7.8	17 422 ± 11 054
Hippuric acid	8	1962 ± 1389	15.7 ± 4.1	95.6 ± 77.8	46 568 ± 30 311
Phloroglucinaldehyde derived					
Ferulic acid^d^	6	87 ± 38	13.3 ± 7.9	96.5 ± 73.4	1816 ± 1054

a Values are expressed as mean ± SEM.

b Metabolite detected in *n* = number of participants.

c PCA-sulfate and VA-sulfate isomers could not be separated sufficiently by HPLC to allow individual quantitation and the values presented are cumulative concentration of both isomers.

d Alternative isomers of ferulic acid include 2-hydroxy-4-methoxycinnamic acid or 4-hydroxy-2-methoxycinnamic acid.

NQ, not quantifiable as compounds remained sufficiently above baseline at 48 h.

C3G reached a maximum serum concentration of 141 nM (Figure 2A), whereas its degradation products, PCA and PGA, were present at 146 nM (Figure 2B) and 582 nM (Figure 3C), respectively, with their *t*_max_ occurring 2–3 h later than that of C3G (Table 2). The metabolites of PCA were the dominant species detected in the serum, with hippuric acid, VA and the B-ring-derived ferulic acid representing the most abundant metabolites. Hippuric acid, PCA-sulfate and ferulic acid had significantly longer elimination half-lives than PCA (Table 2). Eight sulfated and glucuronidated forms of PCA and methylated PCA (VA) were identified within the serum (Figure 2B,C), with the conjugations occurring at both the *meta* and the *para* positions. Of these, VA-sulfate was present in the highest concentration followed by PCA-4-GlcA and PCA-sulfate (Table 2). In contrast, only PGA and one other A-ring-derived metabolite were identified within the serum, namely ferulic acid (Figure 3C; Table 2).

### Urinary elimination

**Figure 4:**
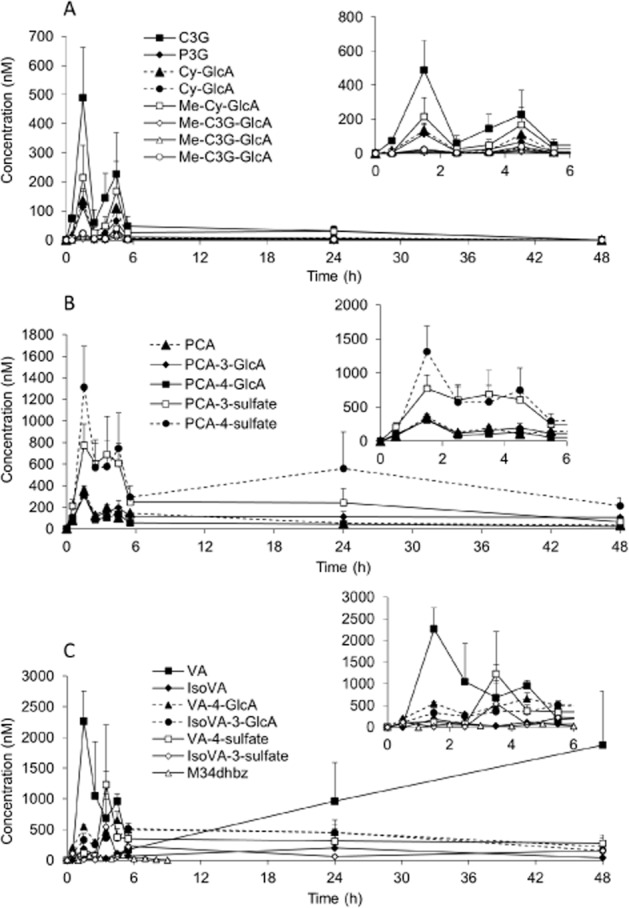
Urinary elimination profiles of (A) C3G and its metabolites, (B) PCA and its metabolites, and (C) methylated PCA and its metabolites in humans after the consumption of 500 mg ^13^C_5_-C3G in eight healthy male participants. All data are mean ± SEM. C3G, cyanidin-3-glucoside; Cy, cyanidin; GlcA, glucuronide; M34dhbz, methyl-3,4-dihydroxybenzoate; Me, methylated; P3G, peonidin-3-glucoside; PCA, protocatechuic acid; VA, vanillic acid.

**Figure 5:**
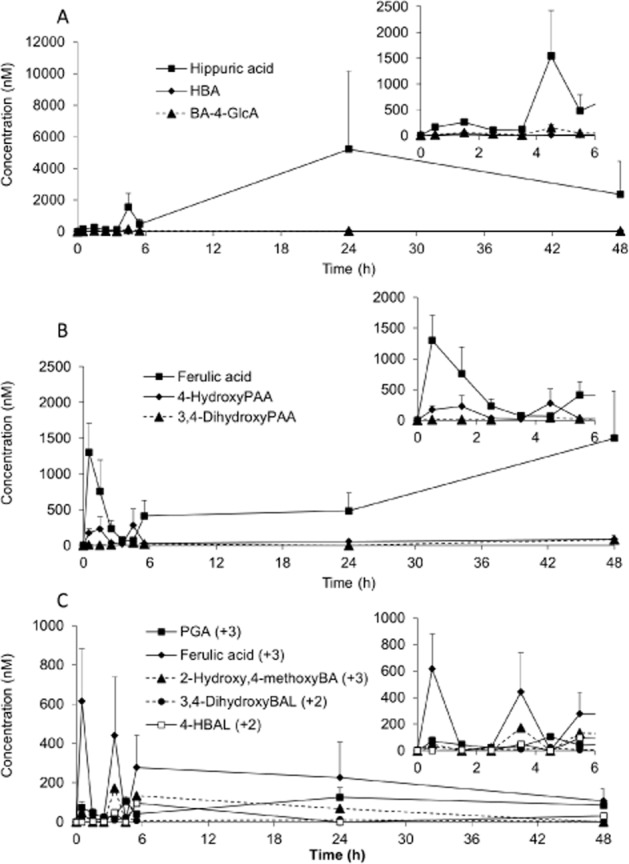
Urinary elimination profiles of (A) benzoic acid metabolites, (B) propenoic acid metabolites, and (C) A-ring-derived and aldehyde metabolites of cyanidin-3-glucoside in humans after the consumption of 500 mg ^13^C_5_-C3G in eight healthy male participants. All data are mean ± SEM. 4-HBAL, 4-hydroxybenzaldehyde; BA, benzoic acid; BAL, benzaldehyde; GlcA, glucuronide; PAA, phenylacetic acid; PGA, phloroglucinaldehyde.

In the urine sample, 31 ^13^C-labelled compounds were identified, comprising C3G, 7 methylated and glucuronidated conjugates of C3G and cyanidin (Cy), PCA, PGA, 19 derivatives of PCA, and 2 derivatives of PGA (Table 3; Figures 4 and 5). Maximum concentrations of C3G and its conjugated derivatives were identified between 1 and 2 h post-consumption (Figure 4A). PCA also reached maximum concentration at 1–2 h (Figure 4B), whereas PGA reached its peak concentration in the urine considerably later (6–24 h) (Figure 5C; Table 3). The phenolic metabolites were excreted in much higher concentrations than the parent anthocyanins, ranging from 24 nM for 3,4-dihydroxybenzaldehyde (Figure 5C) to 5417 nM for hippuric acid (Figure 5A), with peak excretions observed between 6 and 24 h post-consumption (Table 3).

**Table 3:** Urinary recovery of cyanidin-3-glucoside, its degradation products and derived metabolites in humans after the consumption of 500 mg ^13^C-labelled C3G^a^

Metabolite	*n*^b^	Maximum concentration (nM)	Time at maximum concentration (h)^c^	Total recovery (μg)
Parent anthocyanin				
Cyanidin-3-glucoside (C3G)	7	334 ± 145	1–2	120.0 ± 54.1
Cyanidin-glucuronide^d^	5	88 ± 42	1–2	23.9 ± 11.3
Cyanidin-glucuronide^d^	3	19 ± 7	1–2	12.2 ± 6.8
Peonidin-3-glucoside	7	76 ± 43	1–2	22.9 ± 10.6
Methyl-cyanidin-glucuronide^e^	5	206 ± 105	1–2	74.9 ± 36.0
Methyl-C3G-glucuronide^e^	4	6 ± 2	1–2	2.3 ± 1.1
Methyl-C3G-glucuronide^e^	4	14 ± 7	1–2	6.7 ± 3.7
Methyl-C3G-glucuronide^e^	5	20 ± 8	1–2	9.5 ± 5.5
Degradants				
Protocatechuic acid (PCA)	8	337 ± 117	1–2	71.6 ± 20.9
Phloroglucinaldehyde	8	170 ± 42	6–24	66.7 ± 21.3
Protocatechuic acid derived				
Hydroxybenzoic acid^f^	5	49 ± 11	1–2	13.2 ± 3.7
Vanillic acid (VA)	4	3412 ± 312	1–2	960.4 ± 350.0
IsoVA	4	212 ± 130	1–2	79.5 ± 54.2
Methyl-3,4-dihydroxybenzoate	8	108 ± 39	3–4	20.7 ± 8.0
Benzoic acid-4-glucuronide	7	129 ± 47	4–5	53.1 ± 18.1
PCA-3-glucuronide	8	301 ± 72	1–2	197.7 ± 41.2
PCA-4-glucuronide	8	233 ± 65	1–2	100.3 ± 30.2
PCA-3-sulfate	8	1112 ± 318	1–2	322.0 ± 87.3
PCA-4-sulfate	8	1244 ± 333	1–2	492.4 ± 193.0
VA-4-glucuronide	8	762 ± 124	4–5	618.4 ± 109.7
IsoVA-3-glucuronide	8	699 ± 90	5–6	526.8 ± 92.2
VA-4-sulfate	7	1682 ± 899	3–4	449.1 ± 112.4
IsoVA-3-sulfate	5	822 ± 557	3–4	183.2 ± 93.1
Hippuric acid	8	5417 ± 4906	6–24	2415.6 ± 2223.0
Ferulic acid	8	1839 ± 657	24–48	798.2 ± 295.7
4-Hydroxyphenylacetic acid	3	391 ± 113	4–5	50.0 ± 2.0
3,4-Dihydroxyphenylacetic acid	1	82	24–48	28.0
4-Hydroxybenzaldehyde	2	97 ± 94	5–6	10.0 ± 9.0
3,4-Dihydroxybenzaldehyde	6	24 ± 7	0–1	6.3 ± 2.6
Phloroglucinaldehyde derived				
Ferulic acid^g^	8	474 ± 273	0–1	225.1 ± 175.3
2-Hydroxy-4-methoxybenzoic acid	1	172	3–4	64.6

a Values are expressed as mean ± SEM.

b Metabolites detected in *n* = number of participants.

c Samples were pooled across participants urine voids, for *t* = 0–1 h (*n* = 3), *t* = 1–2 h (*n* = 5), *t* = 2–3 h (*n* = 6), *t* = 3–4 h (*n* = 6), *t* = 4–5 h (*n* = 4), *t* = 5–6 h (*n* = 8), *t* = 6–24 h (*n* = 8) and *t* = 24–48 h (*n* = 8).

d Quantified relative to C3G.

e Quantified relative to peonidin-3-glucoside.

f Includes both isomers (3-hydroxybenzoic acid and 4-hydroxybenzoic acid).

g Alternative isomers of ferulic acid include 2-hydroxy-4-methoxycinnamic acid or 4-hydroxy-2-methoxycinnamic acid.

### Recovery of faecal metabolites

A total of 28 ^13^C-labelled compounds were detected in the faeces, including C3G, PCA, PGA, 24 B-ring-derived metabolites and 1 A-ring-derived metabolite (Table 4). B-ring-derived ferulic acid was present at the highest concentration within the faeces, with a maximum recovery of 2373.1 μg at 6–24 h post-bolus, followed by the A-ring-derived ferulic acid (469.7 μg) and PCA (360.9 μg; Table 4).

**Table 4:** Total faecal recovery of cyanidin-3-glucoside its degradation products and derived metabolites in humans between 0–6, 6–24 and 24–48 h after the consumption of 500 mg ^13^C-labelled C3G

Metabolite	Recovery (μg)		
	0–6 h^a^	6–24 h	24–48 h
Parent anthocyanin			
C3G	ND	70.0^b^	19.6^b^
Degradants			
Protocatechuic acid (PCA)	ND	360.9 ± 278.1^c^	221.8 ± 134.9^d^
Phloroglucinaldehyde	ND	2.1 ± 1.3^e^	111.3 ± 78.7^f^
Protocatechuic acid derived			
Hydroxybenzoic acid	ND	ND	22.0 ± 11.6^g^
2,3-Dihydroxybenzoic acid	ND	ND	440.4 ± 381.8^h^
2-Hydroxy-4-methoxybenzoic acid	ND	1.4^b^	273.3 ± 272.7^h^
Vanillic acid (VA)	ND	54.7 ± 1.6^h^	49.3 ± 21.4^g^
IsoVA	ND	11.0^b^	8.5^b^
Methyl-3,4-dihydroxybenzoate	ND	166.6 ± 100.6^g^	92.4 ± 49.4^f^
Methyl vanillate	ND	35.1^b^	ND
Benzoic acid-4-glucuronide	1.5^b^	6.8 ± 3.0^c^	14.8 ± 8.0^i^
PCA-3-glucuronide	ND	22.9 ± 22.0^g^	49.0^b^
PCA-4-glucuronide	ND	16.1 ± 17.5	20.4 ± 16.2^i^
PCA-3-sulfate	0.1^b^	30.0 ± 27.7^h^	11.7 ± 10.5^e^
PCA-4-sulfate	ND	23.0 ± 18.1^g^	12.0 ± 10.2^g^
VA-4-glucuronide	3.6^b^	3.3^b^	9.5 ± 7.9^g^
IsoVA-3-glucuronide	1.8^b^	1.3^b^	12.7 ± 10.9^h^
VA-4-sulfate	0.6^b^	ND	69.0 ± 39.1^g^
IsoVA-3-sulfate	ND	0.6 ± 0.3^h^	176.1 ± 174.7^h^
Hippuric acid	ND	12.0 ± 11.4^g^	27.5 ± 27.4^h^
Caffeic acid	ND	25.2^b^	354.6 ± 125.0^h^
Ferulic acid	ND	2373.1 ± 2905.4^e^	1454.7 ± 1362.0^f^
4-Hydroxyphenylacetic acid	ND	7.3^b^	55.4^b^
3,4-Dihydroxyphenylacetic acid	2.0^b^	10.1 ± 4.0^e^	12.8 ± 7.5^c^
4-Hydroxybenzaldehyde	ND	0.8 ± 0.8^h^	0.1^b^
4-Methoxybenzaldehyde	ND	20.7^b^	ND
3,4-Dihydroxybenzaldehyde	ND	3.4 ± 3.8^h^	5.1 ± 1.8^i^
Phloroglucinaldehyde derived			
Ferulic acid^j^	ND	469.7 ± 687.1^h^	240.8 ± 154.3^c^

a Number of participants providing sample voids at 0–6 h *n* = 2; all participants provided voids for the remaining time points.

Values are expressed as mean ± SEM, where metabolites were detected in ^b^*n* = 1, ^c^*n* = 5, ^d^*n* = 7, ^e^*n* = 4, ^f^*n* = 6, ^g^*n* = 3, ^h^*n* = 2, ^i^*n* = 8 participants.

^j^Alternative isomers of ferulic acid include 2-hydroxy-4-methoxycinnamic acid or 4-hydroxy-2-methoxycinnamic acid.

ND, not detected.

## Discussion and conclusions

Interest in anthocyanins continues to grow as a result of their reported cardiovascular activity (Mink *et al*., 2007; Cassidy *et al*., 2011; 2013). We recently established that anthocyanins are absorbed and metabolized to a greater extent than had been previously reported (Miyazawa *et al*., 1999; Kay *et al*., 2004; 2005; Garcia-Alonso *et al*., 2009). Specifically, using a ^13^C-labelled anthocyanin and isotope-ratio mass spectrometry, we established that a substantial number and amount of breakdown products and conjugates of C3G were absorbed, metabolized and excreted; as represented by the recovery of 12% of the initial dose of ^13^C within the urine sample (Czank *et al*., 2013). The present study indicates that the majority of the label is present in the circulation as lower molecular weight ^13^C-labelled phenolic metabolites of the parent C3G.

By utilizing the targeted labelling of the A- and B-rings of C3G (Figure 1), the present study was able to confirm the pharmacokinetics of the phenolic conjugates of anthocyanins through the identification of ^13^C_5_-, ^13^C_3_- and ^13^C_2_-derived metabolites of the parent anthocyanin. This strategy allowed for the establishment of pharmacokinetic profiles of 17 metabolites in the circulation as well as the elimination of 31 and 28 metabolites in the urine and faeces respectively. To our knowledge, this diversity of anthocyanin metabolites has not been demonstrated previously in humans.

C3G and seven methylated and glucuronidated conjugates of C3G or Cy were identified within the urine sample, reaching cumulative concentrations of 763 nM at 1–2 h post-bolus (Figure 4A), which is in accordance with the previous studies (Kay *et al*., 2004; Manach *et al*., 2005; McGhie and Walton, 2007). However, the parent anthocyanin only represented 2% of the total metabolites found in the circulation and was only present for a relatively short period of time (*t*½, 0.4 h; Table 2), thus suggesting that anthocyanin bioactivity is likely to be mediated by high concentrations of its phenolic intermediates as opposed to the parent structure.

It is well established that C3G degrades to PCA and PGA when incubated at neutral pH (Kay *et al*., 2009; Woodward *et al*., 2009) and this is corroborated by the present data, as PCA (*t*_max,_ 3.3 h; Figure 2B) and PGA (*t*_max,_ 2.8 h; Figure 3C) were some of the earliest phenolic metabolites identified in the circulation (Table 2). In the present study, PCA was observed at maximum concentrations of 147 nM, thus suggesting that it is not a major metabolite of anthocyanins. The A-ring-derived degradation product, PGA, was present at concentrations greater than either C3G or PCA in the serum (Table 2 and Figure 3C). However, it was not detected in urine at any appreciable concentration, suggesting that it was further metabolized prior to elimination (Table 3 and Figure 5C). Multiple phase II metabolites of PCA were detected in early (from 0.5 h to 1 h) serum samples, suggesting that the degradation of anthocyanins *in vivo* is swiftly followed by further biotransformation.

Hippuric acid was identified as the major metabolite of anthocyanins in the present study, reaching a maximum concentration of 1962 nM in the serum (Table 2). Hippuric acid has been speculated to be a common metabolite for many flavonoids (Pero, 2010) but it is a challenging metabolite to identify without the use of an isotope label due to its high background levels derived from other dietary and endogenous (i.e. protein metabolism and amino acid catabolism) sources, where it is reported to reach 1–2 mM concentrations in human urine (Toromanović *et al*., 2008; Pero, 2010). The detection of ^13^C_2_-labelled hippuric acid in the present study indicates that PCA and its conjugates are likely further metabolized to form BA, which is conjugated with glycine to form hippuric acid, or alternatively, formed from the alpha-oxidation and dehydroxylation of hydroxyphenylacetic acids (Mullen *et al*., 2008). This suggests that glycine conjugation is a key metabolic process responsible for the clearance of phenolic metabolites of C3G from the body.

VA, the methylated metabolite of PCA, was also present in the serum in high concentrations (Table 2 and Figure 2C); however, it was only identified in the serum in two participants. Sulfation appeared to be the preferential metabolic process for the clearance of PCA, as PCA-sulfate (Figure 2B) and VA-sulfate (Figure 2C) were detected in much higher concentrations than their unconjugated or GlcA counterparts. This pattern of elimination of PCA metabolites in the serum was mirrored in the urine samples, with VA detected at the highest concentration, followed by *meta* and *para* sulfated and glucuronidated conjugates of PCA and VA (Table 3). Di-methylation of PCA was also observed by the presence of methyl-3,4-dihydroxybenzoate and methyl vanillate; however, methyl vanillate was only detected in the faeces (Table 4), suggesting that di-methylation of PCA results in biliary elimination, or more likely, is a product of microbial metabolism. Therefore, a primary route of metabolism and subsequent elimination of C3G appears to be through degradation to PCA, followed by rapid methylation of the catechol group to form VA, and subsequent sulfate and GlcA conjugation, thus increasing its polarity and elimination from the body. This finding is supported by previous studies feeding catechin to rats and guinea pigs, where unconjugated metabolites represented only 3.5–4.4% of the recovered phenolic metabolites (Das and Griffiths, 1969). Minimal amounts of phase I metabolites of PCA were also detected in this study, including 3,4-dihydroxybenzaldehyde (Figure 5C) and hydroxybenzoic acid (Figure 5A) within urine and 4-HBAL within serum (Figure 3C) and urine (Figure 5C), suggesting that phase I metabolism may not be a highly utilized pathway for anthocyanin metabolism.

Many metabolites of PCA in the present study appeared to follow biphasic serum kinetics, displaying an initial serum peak between 0 and 5 h followed by a second peak between 6 and 48 h (Figures 2 and 3). Biphasic absorption or elimination of delphinidin-3-glucoside has previously been reported in rats, with maximum plasma concentrations reported at 15 and 60 min (Ichiyanagi *et al*., 2004) and also within human studies feeding flavonoids, where isoflavonoids and metabolites including caffeic acid, ferulic acid and ferulic acid-sulfate were reported to follow biphasic plasma kinetic patterns (Anupongsanugool *et al*., 2005; Wittemer *et al*., 2005; Azzini *et al*., 2007; Stalmach *et al*., 2009; Rodriguez-Mateos *et al*., 2013). The biphasic profiles most likely result from metabolism occurring in multiple tissues, including the liver and at different sites within the gastrointestinal tract.

The route of elimination of the A-ring-derived metabolites is less clear. A total of three A-ring-derived metabolites were identified in the serum, urine and faecal samples (PGA, 2-hydroxy-4-methoxybenzoic acid and a compound putatively identified as ferulic acid). The metabolite tentatively identified as ferulic acid matched the molecular weight (+3), fragmentation pattern and had a retention time similar to the ferulic acid standard (Table 1); however, the exact structural conformation of ^13^C_3_-ferulic acid cannot be established with certainty, as other probable isomers could result from the methylation of the A-ring hydroxyl to form either 2-hydroxy-4-methoxycinnamic acid or 4-hydroxy-2-methoxycinnamic acid. We are not aware of previous studies reporting these metabolites and more work is required to confirm their exact structural identities. In addition, A-ring-derived 3,4-dihydroxybenzaldehyde and methyl 3,4-dihydroxybenzoate were tentatively identified within post-bolus samples; however, due to their low concentrations, they were not included in the present analyses. At present, little is known about the metabolism of PGA and further studies are required to delineate the mechanisms involved in its metabolism and clearance.

Colonic metabolism has long been speculated to be a major contributor to the overall metabolism of anthocyanins (Williamson and Clifford, 2010; Cardona *et al*., 2013) and our ^13^C-labelled C3G study reported 32 ± 6% of the recovered ^13^C-label in faeces (Czank *et al*., 2013). It has been proposed that phenylpropenoic acids arise from C3G as a result of bacterial cleavage of the C-ring in the colon (Forester and Waterhouse, 2008; Gonzalez-Barrio *et al*., 2011), which is supported by the detection of caffeic acid and its methyl metabolite, ferulic acid, within the faeces in the present investigation. The absence of caffeic acid in the serum and urine despite its abundance within the faeces (Table 4) suggests that the catechol group of caffeic acid is rapidly methylated to yield ferulic acid, either during intestinal absorption, prior to entry into the systemic circulation or by the liver. The rapid metabolism of caffeic acid has been observed in previous phenolic acid intervention studies, where caffeic acid is reported at significantly lower concentrations than its methyl, glucuronyl and sulfate conjugates (Nardini *et al*., 2006). In addition, ferulic acid was detected within the serum and urine samples within 4 h of consumption, indicating that ferulic acid may, in part, be formed proximal to the colon (i.e. the middle and lower small intestine).

**Figure 6:**
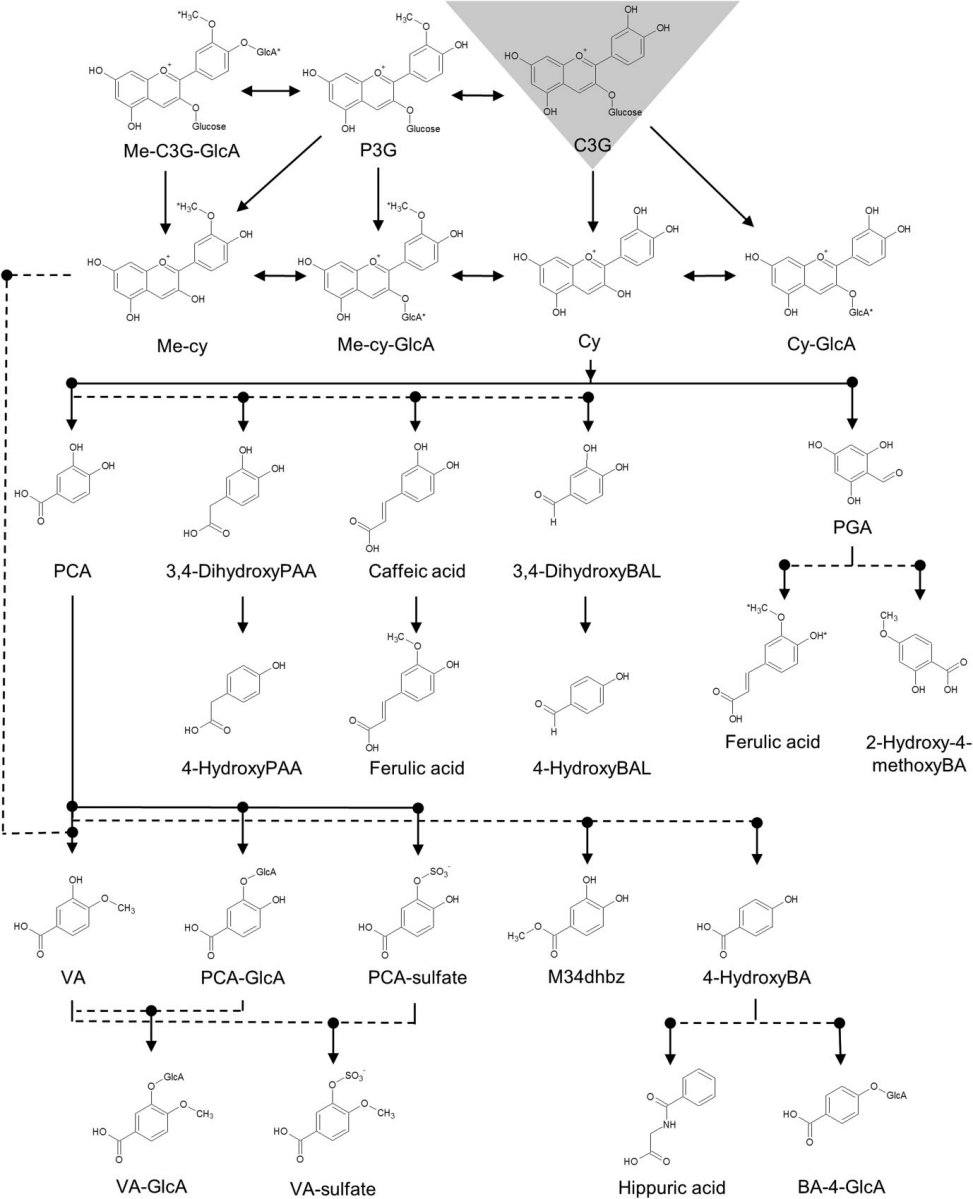
Proposed pathway for the metabolism of cyanidin-3-glucoside in humans. *Conjugates represent unknown structural position. BA, benzoic acid; BAL, benzaldehyde; C3G, cyanidin-3-glucoside; Cy, cyanidin; GlcA, glucuronide; Me, methoxy; M34dhbz, methyl-3,4-dihydroxybenzoate; P3G, peonidin-3-glucoside; PAA, phenylacetic acid; PGA, phloroglucinaldehyde.

On the basis of the findings of this study, the metabolism of anthocyanins can be summarized as undergoing multiple biotransformations (Figure 6). The parent compound appears to undergo methylation and glucuronidation to some extent; however, it primarily undergoes degradation, followed by significant phase II conjugation, particularly methylation and sulfation. Given the early timing and high concentrations of PCA and PGA observed in this study, it is clear that C3G spontaneously degrades to PCA and PGA in the small intestine and circulation; however, colonic metabolism is also likely to play a significant role. Dehydroxylation by colonic bacteria, to form hydroxybenzoic acid, followed by conjugation with glycine to form hippuric acid, appears to be the definitive elimination pathway. Finally, the metabolism of PGA to phenylpropenoic acids, which are similar to, or include, ferulic acid, may be responsible for the detoxification and elimination of PGA.

The biological activities of the parent anthocyanins has been previously reported (Xu *et al*., 2004; Bell and Gochenaur, 2006; Kim *et al*., 2006; Nizamutdinova *et al*., 2009; Zhu *et al*., 2011); however, the activities of the phenolic metabolites identified in the present study are relatively unknown. Many of the metabolites identified share structural similarities with known bioactives such as apocynin, an inhibitor of NADPH oxidase (Kim *et al*., 2011), and salicylic acid, known for its anti-inflammatory activity (Vane and Botting, 2003). Of the limited information available for many of the presently identified metabolites, PCA has been shown to reduce vascular cell adhesion molecule-1 (VCAM-1) protein and mRNA in TNF-α-induced mouse aortic endothelial cells (Wang *et al*., 2010). In addition, plasma VCAM-1 levels were reduced in mice fed PCA (Wang *et al*., 2010), whereas vanillic acid fed mice showed reductions in plasma IL-6 (Kim *et al*., 2010). Moreover, in LPS-induced RAW 264.7 cells, PCA has been shown to suppress the production of TNF-α, IL-1β, NO, PGE_2_ and the gene expression of NOS and COX-2 (Min *et al*., 2010). Together, these studies suggest that the bioactivity of anthocyanins is likely attributed to their phenolic metabolites, which may have both vascular and anti-inflammatory activity. However, a great deal of work is required to confirm the bioactivities of the extensive numbers of metabolites identified herein.

The major limitation of the presently employed analytical methodology is its targeted approach, which relies on pure standards and MRM for identification. This approach is highly sensitive; however, it does not allow for the identification of unknown/untargeted metabolites. Additional synthesis of the speculated metabolites is necessary to confirm their identities. In addition, alternative spectroscopy techniques such as GC-MS/MS and high resolution time-of-flight MS would aid in identifying further metabolites not captured by the present methodology. As many of the metabolites did not completely returned to baseline by 48 h, a longer blood, urine and faecal sampling time may have increased the recovery of metabolites. Despite these limitations, this human study has demonstrated for the first time that a wide array of metabolites are recovered by the described extraction and detection methods and these data represent a considerable advancement in our understanding of anthocyanin ADME.

In conclusion, the extensive number of anthocyanin metabolites identified, the appearance of multiple peaks across the 48 h time period and the wide range of their elimination half-lives suggest that the clearance of anthocyanins involves multiple processes, including enterohepatic recirculation, hepatic recycling and microbial metabolism, with an extended period of intestinal absorption from both the small and the large bowel. This study provides new insights into the metabolism of anthocyanins, which should inform the design of future clinical studies exploring the bioactivity of these potentially important dietary compounds.

## Abbreviations

ADME: absorption, distribution, metabolism and elimination
BA: benzoic acid
C3G: cyanidin-3-glucoside
Cy: cyanidin
GlcA: glucuronide
4-HBAL: 4-hydroxybenzaldehyde
P3G: peonidin-3-glucoside
PCA: protocatechuic acid
PGA: phloroglucinaldehyde
VA: vanillic acid
